# Potential of Multiplex Polymerase Chain Reaction Performed on Protected Telescope Catheter Samples for Early Adaptation of Antimicrobial Therapy in ARDS Patients

**DOI:** 10.3390/jcm11154366

**Published:** 2022-07-27

**Authors:** Keyvan Razazi, Flora Delamaire, Vincent Fihman, Mohamed Ahmed Boujelben, Nicolas Mongardon, Ségolène Gendreau, Quentin de Roux, Nicolas de Prost, Guillaume Carteaux, Paul-Louis Woerther, Armand Mekontso Dessap

**Affiliations:** 1 AP-HP (Assistance Publique-Hôpitaux de Paris), Hôpitaux Universitaires Henri Mondor, DMU Médecine, Service de Médecine Intensive Réanimation, CHU Henri Mondor, 51, Av. de Lattre de Tassigny, CEDEX, 94010 Créteil, France; flora.delamaire@chu-rennes.fr (F.D.); mohamedahmed.boujelben@aphp.fr (M.A.B.); segolene.gendreau@aphp.fr (S.G.); nicolas.de-prost@aphp.fr (N.d.P.); guillaume.carteaux@aphp.fr (G.C.); armand.dessap@aphp.fr (A.M.D.); 2 Faculté de Médecine de Créteil, IMRB, GRC CARMAS, Université Paris Est Créteil, 94010 Créteil, France; 3 AP-HP (Assistance Publique-Hôpitaux de Paris), Hôpitaux Universitaires Henri Mondor, Département de Prévention, Diagnostic et Traitement des Infections, Unité de Bactériologie, 94010 Créteil, France; vincent.fihman@aphp.fr (V.F.); paul-louis.woerther@aphp.fr (P.-L.W.); 4 EA 7380 Dynamyc, UPEC, Ecole Nationale Vétérinaire d’Alfort, USC Anses, 94000 Créteil, France; 5 AP-HP Service d’Anesthésie-Réanimation Chirurgicale, DMU CARE, DHU A-TVB, Hôpitaux Universitaires Henri Mondor, 94010 Créteil, France; nicolas.mongardon@aphp.fr (N.M.); quentin.deroux@aphp.fr (Q.d.R.); 6 U955-IMRB, Equipe 03 “Pharmacologie et Technologies Pour les Maladies Cardiovasculaires (PROTECT)”, Inserm, Université Paris Est Créteil (UPEC), Ecole Nationale Vétérinaire d’Alfort (EnVA), 94700 Maisons-Alfort, France; 7 Faculté de Santé, Université Paris Est Créteil, 94010 Créteil, France; 8 INSERM, Université Paris Est, Unité U955, 94010 Créteil, France

**Keywords:** ventilator-associated pneumonia, COVID-19, multiplex PCR, antibiotic stewardship, superinfection, coinfection

## Abstract

Background: Diagnosis of co/superinfection in patients with Acute Respiratory Distress Syndrome (ARDS) is challenging. The FilmArray Pneumonia plus Panel (bioMérieux, France), a new rapid multiplex Polymerase Chain Reaction (mPCR), has never been assessed on a blinded protected telescope catheter (PTC) samples, a very common diagnostic tool in patients under mechanical ventilation. We evaluated the performance of mPCR on PTC samples compared with conventional culture and its impact on antibiotic stewardship. Methods: Observational study in two intensive care units, conducted between March and July 2020, during the first wave of the COVID-19 pandemic in France. Results: We performed 125 mPCR on blinded PTC samples of 95 ARDS patients, including 73 (77%) SARS-CoV-2 cases and 28 (29%) requiring extracorporeal membrane oxygenation. Respiratory samples were drawn from mechanically ventilated patients either just after intubation (*n* = 48; 38%) or later for suspected ventilator-associated pneumonia (VAP) (*n* = 77; 62%). The sensitivity, specificity, positive, and negative predictive values of mPCR were 93% (95% CI 84–100), 99% (95% CI 99–100), 68% (95% CI 54–83), and 100% (95% CI 100–100), respectively. The overall coefficient of agreement between mPCR and standard culture was 0.80 (95% CI 0.68–0.89). Intensivists changed empirical antimicrobial therapy in only 14% (18/125) of cases. No new antibiotic was initiated in more than half of the CAP/HAP pneumonia-suspected cases (*n* = 29; 60%) and in more than one-third of those suspected to have VAP without affecting or delaying their antimicrobial therapy. Conclusions: Rapid mPCR was feasible on blinded PTC with good sensitivity and specificity. New antibiotics were not initiated in more than half of patients and more than one-third of VAP-suspected cases. Further studies are needed to assess mPCR potential in improving antibiotic stewardship.

## 1. Introduction

Most patients in the intensive care unit (ICU) receive antimicrobial therapy [1]. Patients with SARS-CoV-2 infection complicated with Acute Respiratory Distress Syndrome (ARDS) are at higher risk of developing ventilator-associated pneumonia (VAP) [2,3]. Diagnosis of VAP in ARDS patients is challenging given the poor accuracy of chest X-rays to detect new infiltrates [4]. Although the deterioration in oxygenation and the arousal of systemic inflammatory responses are mostly incurred by noninfectious events in mechanically ventilated patients, they are seen as signs of a new infection requiring broad-spectrum antibiotics [5]. Identifying the causative microorganisms helps implement a target antibiotic therapy, but the turnaround time from microbiological sampling to obtaining culture results takes at least 48 h. The FilmArray^®^ Pneumonia plus Panel (bioMérieux, Marcy l’Étoile, France) is a rapid microbiological diagnostic assay based on nested multiplex PCR (mPCR) method. The test is a semi-quantitative approach to detect a panel of 15 bacteria and a qualitative approach to detect three atypical bacteria, nine viruses, and seven antibiotic resistance genes within 1.5 h from sputum, endotracheal aspirate, or bronchoalveolar lavage (BAL) specimens draw. The performance of mPCR has not been assessed on samples of a protected telescopic catheter (PTC), although the latter is the most commonly employed tool to diagnose nosocomial pneumonia in many countries, including France [6], and its utility is equivalent to that of bronchoalveolar lavage in Europe [7].

During the first wave of COVID-19, the French Society of Microbiology issued recommendations to limit the exposure of laboratory technicians to potentially SARS-CoV-2 contaminated samples [8]. For such, we abandoned smearing of respiratory specimens and focused on a rapid molecular diagnostic method to anticipate culture results. The objective of the present study was to evaluate (i) the performance of mPCR on PTC and (ii) whether it helped to initiate or early adjust empirical antimicrobial therapy in critically ill patients during the first outbreak of COVID-19, when direct smear examination was not advised, in two Intensive Care Units (ICUs) of a tertiary university hospital.

## 2. Materials and Methods

We conducted a monocenter retrospective analysis on 95 critically ill patients admitted to two ICUs of Henri-Mondor University Hospital between March and July 2020. During that period and in compliance with the recommendations of the French Society of Microbiology [8], Gram staining of respiratory samples was discouraged to spare the laboratory staff the risk of aerosolization of SARS-CoV-2. Instead, mPCR was performed at the physician’s request, following local guidelines, if bacterial co/superinfection was suspected. For any suspected case of pneumonia, mPCR was requested if one of the following three conditions was present: (i) admission of more than 48 h without microbiological documentation on a pulmonary specimen of less than 48 h; (ii) respiratory or hemodynamic impairment after more than 72 h of prior antibiotic therapy; (iii) risk factors for multidrug-resistant organism infection (*P. aeruginosa* or extended-spectrum β-lactamase producing Enterobacteriaceae [ESBL-PE] known colonization, chronic obstructive pulmonary disease, bronchial dilatation, dialysis, solid organ transplant recipients).

### 2.1. VAP Definition

VAP was suspected if any of its classical criteria happened 48 h or more after mechanical ventilation initiation: new or worsening infiltrates on chest X-ray, systemic signs of infection (new-onset fever, leukocytosis or leucopenia, increased need for vasopressors to maintain blood pressure), purulent secretions, and impaired oxygenation [9]. VAP diagnosis was confirmed on quantitative cultures of lower respiratory tract secretions sampled before administering new antibiotics using a blinded or bronchoscopy-driven PTC (10^3^ colony forming units/mL). PTC was performed as previously described (combicath^®^ Prodimed, Le Plessis Bouchard, France) [10]. Of note, mPCR reports bacteria only if more than 10^4^ copies per ml were present, so all positive PCR indicated that the reportable threshold was attained. According to guidelines, mPCR was not considered for VAP definition.

### 2.2. Culture

Briefly, the PTC specimen was manually liquefied using a volume-to-volume dilution with mucolytic SL solution (Copan, Brescia, Italy) and then inoculated onto trypticase soy (TS; Oxoid, Dardilly, France), Drigalski agar incubated in aerobic incubators, PolyViteX-supplemented chocolate agar and CNA agar plates (bio-Merieux) incubated in 5% CO2 incubators, and COH agar incubated in anaerobic conditions. All agar media were incubated at 35 °C for 48 h. Hospital-acquired pneumonia (HAP) was defined as a lower respiratory tract infection developed after 48 h of hospital admission. Pneumonia developed earlier than that should be considered community-acquired pneumonia (CAP). Bacterial identification was not necessary for CAP and HAP definitions. However, for both community and hospital-acquired pneumonia, if the patient had already been on antibiotics at the time of sampling, the bacteria with a count between 10^2^ and 10^3^ CFU/mL were studied. Matrix-Assisted Laser Desorption/Ionization-Time-Of-Flight mass spectrometer (Microflex LT, Bruker Daltonics, Bremen, Germany) was used for bacterial identification. Antibiotic susceptibility testing (AST) was performed using the disc diffusion method on Mueller–Hinton media (Bio-Rad, Marnes-la-Coquette, France) on colonies isolated after the primary culture, according to EUCAST recommendations (www.eucast.com (accessed on 1 January 2020)). ESBL in Enterobacteriales and methicillin resistance in *staphylococci* were determined phenotypically on AST.

### 2.3. mPCR

FilmArray^®^ Pneumonia plus panel was implemented according to the manufacturer’s instructions using 200 µL of the mucolytic SL-diluted solution (Copan) as a sample for the pouch-based mPCR with FilmArray Torch instrument [11]. Intensivists could have the results of mPCR within two hours of receiving the sample at the laboratory, 24/7.

### 2.4. Antibiotic

De-escalation was defined, according to previous studies, as discontinuation of any companion drug and/or switching from the pivotal drug to a narrower-spectrum antibiotic according to Weiss’classification [12]. Adequate antimicrobial therapy was defined as the administration of at least one agent active in-vitro on the causative pathogens.

### 2.5. Statistical Analysis

Conventional cultures were the reference standard in this study; i.e., microorganisms identified only by mPCR and not by conventional culture were marked as false positives. Viruses, *Chlamydophila pneumoniae*, and *Mycoplasma pneumoniae* were excluded from analysis since they are not routinely sought in clinical microbiology. Agreement between the two methods was assessed by calculating Cohen’s k coefficient. The diagnostic value of mPCR was assessed using the conventional culture as the reference method, only for the microorganisms covered by the mPCR panel. Analyses were performed using the R 3.1.2 package mstate [13] (R Foundation for Statistical Computing, Vienna, Austria).

## 3. Results

### 3.1. Patients

Over the study period, 95 patients were included, of whom 73 (77%) had SARS-CoV-2 infection. Patient characteristics and ICU data are presented in Table 1. All enrolled patients fulfilled ARDS criteria, and 28 (29%) were on extracorporeal membrane oxygenation (ECMO). All respiratory samples were drawn from patients under mechanical ventilation. mPCR was performed on 125 PTC samples, of which 48 suspected CAP or HAP immediately after intubation, and 77 suspected VAP developed after a median of 8 [5–17] days of mechanical ventilation. A total of 73 (58%) samples were taken from patients who were in shock (required catecholamine).

### 3.2. Culture and mPCR

The conventional culture isolated at least one bacteria species in 34 (27%) samples (from a total of 41 bacteria, of which 33 exceeded the reportable threshold and eight were below it). mPCR detected at least one bacteria species in 31 (25%) samples (from a total of 41 bacteria, all of which were above the threshold, as per the assay configuration).

**Figure 1:**
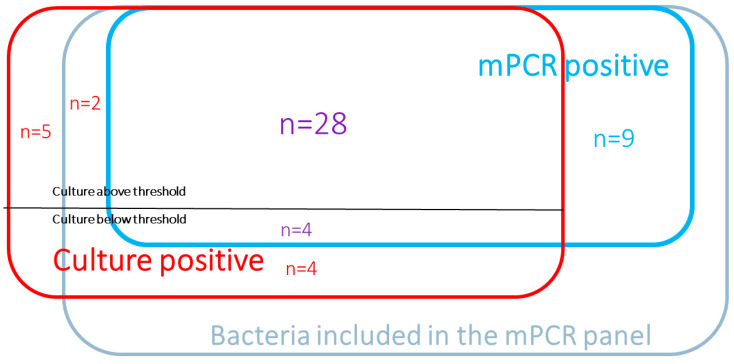
Bacteria distribution according to culture and mPCR results.

Overall, 48 bacteria were identified above the threshold, including 28 by culture and mPCR, 13 by mPCR only, and seven by culture only (Figure 1).

The 13 bacteria identified by mPCR (in 11 patients) included four bacteria which were equally isolated by culture but below the threshold [*Escherichia coli* (*n* = 2), *Klebsiella aerogenes* (*n* = 1), and *Enterobacter cloacae* (*n* = 1)], and nine bacteria that the culture failed to detect [(*Staphylococcus aureus* (*n* = 3), *Streptococcus agalactiae* (*n* = 2), *Streptococcus pneumoniae* (*n* = 1), *Haemophilus Influenzae* (*n* = 1), *Pseudomonas aeruginosa* (*n* = 1), and *Legionella pneumophila* (*n* = 1)]. Four of those nine bacteria were identified by culture in previous samples (*Staphylococcus aureus* (*n* = 1); *Streptococcus agalactiae* (*n* = 1); *Pseudomonas aeruginosa* (*n* = 1); and *Legionella pneumophila* (*n* = 1). In seven of the thirteen mPCR-detected cases, the patients had already been on an antibiotic active against the causative bacteria at the time of respiratory sampling.

The seven bacteria identified above the threshold by culture included five bacteria not spanned by the panel (*Burkholderia cepacia*, *Citrobacter* spp., *Staphylococcus lugdunensis, Corynebacterium accolens*, and *Enteroccocus faecalis*) and two covered by the panel yet only revealed by conventional culture (*Klebsiella aerogenes* and *Staphylococcus aureus*).

Overall, eight bacteria were identified below the threshold, four by both methods (see above), and four by culture alone (*Pseudomonas aeruginosa*, *Proteus mirabilis, Escherichia coli,* and *Citrobacter freundii*).

Five CAP/HAP were documented (two were mPCR and culture positive, two were only culture positive, and one was only mPCR positive), and nineteen VAP were diagnosed (i.e., with culture ≥10^3^ CFU/mL).

### 3.3. Diagnostic Value of mPCR

The sensitivity, specificity, positive, and negative predictive values of mPCR to identify bacteria retrieved by culture above the threshold were 93% (95% CI 84–100), 99% (95% CI 99–100), 68% (95% CI 54–83), and 100% (95% CI 100–100), respectively (Table 2). The sensitivity, specificity, positive, and negative predictive values of mPCR to identify bacteria retrieved by culture irrespective of the threshold were 86% (95% CI 75–98), 100% (95% CI 99–100), 78% (95% CI 65–91), and 100% (95% CI 100–100), respectively (Table S1). The overall coefficient of agreement between mPCR and culture was 0.80 (95% CI 0.69–0.90) for a positive culture above the threshold (Table 2) and 0.83 (95% CI 0.74–0.92) for a positive culture irrespective of the threshold. These results were similar among patients with or without SARS-CoV-2 infection (Table S2).

### 3.4. Antibiotic Resistance and Therapy

mPCR test detected eight antibiotic-resistant gene carriers, including six true positives (four *bla*_CTX-M_, one *bla*_OXA 48_, and one *bla*_NDM_), and two false positives for Methicillin resistance of *Staphylococcus aureus* (*mecA/C* with MREJ).

Based on the results of mPCR alone, the intensivists did not initiate any antibiotic therapy in ten (8%) cases, did not change ongoing antibiotic therapy initiated before (48 cases, 38%) or after (49 cases; 39%), suspecting pneumonia in them, but did modify empirical therapy in 18 (14%) cases (de-escalated in seven and escalated in eleven (Table 2). No new antibiotic was initiated in more than half of the CAP/HAP pneumonia-suspected cases (60%; *n* = 29) and more than one-third of VAP-suspected patients (38%; *n* = 29).

In the CAP/HAP-suspected cases, the positive mPCR results led to de-escalations (*n* = 3/3, 100%) (to a narrower spectrum), whereas the negative results led mainly to no change in antibiotic therapy (*n* = 44/45; 98%) (Table 3). In the VAP-suspected cases, the positive mPCR results more often led to no change in antibiotic (*n* = 16/28; 57%) than to escalation (*n* = 11/28; 39%), whereas the negative results more often led to no change in antibiotic (*n* = 47/49; 96%) than to de-escalation (*n* = 2/49; 4%) (to a narrower spectrum) (Table 3). All nineteen VAP cases received adequate antimicrobial therapy after seeing their mPCR results.

## 4. Discussion

We herein showed that mPCR has an overall good performance upon using PTC as the diagnostic sampling technique. mPCR seems to be a useful tool to guide antibiotic stewardship in ARDS patients in the absence of direct smear examination. No new antibiotic was initiated in nearly half of the patients suspected to have pneumonia and more than one-third of those suspected to have VAP.

mPCR performance with PTC is consistent with the findings of previous studies that examined mPCR with other sampling methods where a direct examination was available [14,15]. This result is important for daily practice since PTC is the main diagnostic sampling technique in intubated patients in France [6].

According to the guidelines, antibiotic therapy should be started as soon as possible in severe cases suspected to have VAP or HAP. However, in the recent surviving sepsis campaign guidelines, in adults with possible sepsis and not in shock, a time-limited, rapid investigation is advised [16]. Moreover, recent studies have shown that an antibiotic stewardship program that initiates antibiotic therapy only after microbiological identification, except for septic shock or severe ARDS, is safe [17]. As evidenced, mPCR results were obtained a couple of hours after sampling; therefore, intensivists could wait for such results to optimize antibiotic prescription. Despite ARDS status and the high proportion of patients requiring catecholamine and ECMO, the very good negative predictive value of mPCR spared patients unnecessary broad-spectrum antibiotics. When new antibiotic courses were initiated, a negative mPCR result did not incite doctors to withdraw the antibiotic. Their reasoning was the seriousness of the case, or because the potential clinically relevant bacteria were not covered by the PCR panel, and the fact that stopping treatment without available culture is unusual for intensivists, as previously shown in another monocentric study on COVID-19 patients [18]. Moreover, in some cases, bacteria were detected by culture and not by mPCR [14]. Notwithstanding, the panel of targets does not include some clinically relevant Enterobacteriales (especially *Citrobacter* spp., *Hafnia alvei*, and *Morganella morganii*) and some opportunistic non-fermentative Gram-negative bacteria species (such as *Stenotrophomonas maltophilia and Achromobacter* spp., *Burkholderia cepacia)*. For all those reasons, in a strongly suspected case of VAP, albeit with negative mPCR, empiric broad-spectrum antimicrobial therapy must still be administered.

A potential concern of PCR-based methods is that they may detect residual nucleic acids rather than viable material. In our study, four samples were mPCR-positive and culture-negative, and showed the same microorganisms that had already been found in the culture of previous samples, i.e., mPCR detected their residue [15]. Detecting residual materials can be advantageous for patients previously treated with antibiotics (e.g., CAP). Still, it can also lead to overdiagnosis and over-prescription of antibiotics since fourteen mPCR-positive samples were culture-negative (below the threshold). Additionally, we showed, in accordance with previous studies, that false positives of *mecA/C* and MREJ were not exceptional [14,15].

Our study has the following limitations. First, it was conducted in a single center on ARDS patients. The number of patients was relatively small. A higher number of patients could have increased the reproducibility and statistical relevance of this study. However, the rate of documented CAP/HAP is consistent with that found in multicenter studies of coinfection at ICU admission [19,20]. Similarly, the rate of documented suspected VAP is in accordance with previous reports [5]. Second, the clinical decisions of ICU doctors were shared with multidisciplinary staff (infectious diseases specialists, microbiologists, and pharmacists), and mPCR was performed 24/7 in a microbiological lab geographically close to the two ICUs of our hospital. Thus, extrapolation to other centers with a different local organization and/or epidemiology might be questionable. Third, the impact of mPCR on patient outcomes could not be assessed as no control group was included.

## 5. Conclusions

We herein report the feasibility of mPCR on PTC samples. mPCR offers good potential to improve antimicrobial stewardship. A solid step that opens the door widely for randomized control trials where patients either receive treatment guided by results of the mPCR test or standard care.

## Figures and Tables

**Table 1:** Characteristics of patients.

Clinical Characteristics and Comorbidities	Patients *n* = 95
Age, years, median [IQR]	60 [52–71]
Male gender, *n* (%)	79 (80%)
SAPS II at ICU admission, median [IQR]	38 [30–50]
Charlson Comorbidity index, median [IQR]	3 [2–5]
Diabetes mellitus, *n* (%)	40 (40%)
Congestive heart failure (NYHA 3–4), *n* (%)	6 (6%)
COPD, *n* (%)	9 (9%)
Immunosuppression condition, *n* (%)	21 (22%)
**Organ failures and outcome**	
ARDS	95 (100%)
Extracorporeal membrane oxygenation	28 (29%)
Dialysis	42 (44%)
White blood cell count (×10^9^/L)	11.4 [8.7–15.9]
C-Reactive Protein, mg/L	143 [91–216]
Procalcitonin, µg/L	1.0 [0.3–4.8]
Death in ICU	42 (44%)

SAPS, simplified acute physiologic score; ICU, intensive care unit; ARDS, acute respiratory distress syndrome.

**Table 2:** Analytical performance of BioFire^®^ FilmArray^®^ Pneumonia plus Panel compared with culture, accounting for microbiological thresholds.

Bacterial Target	No. of Specimens				mPCR Performance				Cohen’s Kappa Coefficient
	Culture+/FA-PP+	Culture+/FA-PP−	Culture−/FA-PP+	Culture−/FA-PP−	Se (95% CI), %	Sp (95% CI), %	PPV (95% CI), %	NPV (95% CI), %	
***Acineterobacter calcoaceticus-baumannii* complex**	0	0	0	125	NA	100	NA	100	
***Enterobacter cloacae* complex**	2	0	1	122	100	99	67	100	
***Escherichia*** ***coli***	2	0	2	121	100	98	50	100	
***Haemophilus influenzae***	0	0	1	124	NA	99	0	100	
***Klebsiella aerogenes***	2	1	1	121	67	99	99	98	
***Klebsiella*** ***oxytoca***	0	0	0	125	NA	100	NA	100	
***Klebsiella*** ***pneumoniae* group**	0	0	0	125	NA	100	NA	100	
***Moraxella*** ***catarrhalis***	1	0	0	124	100	100	100	100	
***Proteus*** **spp.**	2	0	0	123	100	100	100	100	
***Pseudomonas*** ***aeruginosa***	11	0	1	113	100	99	92	99	
***Serratia marcescens***	1	0	0	124	100	100	100	100	
***Streptococcus*** ***pneumoniae***	1	0	1	123	100	99	50	100	
***Staphylococcus aureus***	5	1	3	116	83	97	63	99	
***Streptococcus*** ***pyogenes***	0	0	0	125	NA	100	NA	100	
***Streptococcus*** ***agalactiae***	0	0	2	123	NA	98	0	100	
***Legionella pneumophila***	1	0	1	123	100	99	50	100	
**TOTAL**	**28**	**2**	**13**	**1957**	**93 [84–100]**	**99 [99–100]**	**68 [54–83]**	**100 [100]**	**0.8 [0.68–0.89]**

Se, sensitivity; Sp, specificity; PPV, positive predictive value; NPV, negative predictive value; FA-PP, FilmArray^®^ Pneumonia plus Panel.

**Table 3:** Impact of mPCR results on antibiotic prescription (*n* =125).

		Suspected CAP/HAP Cases (*n*= 48)		Suspected VAP Cases (*n*= 77)	
		mPCR − (*n* = 45)	mPCR + (*n* = 3)	mPCR − (*n* = 49)	mPCR + (*n* = 28)
**Antibiotic modification after mPCR**		**1**	**3**	**2**	**12**
**De-escalation**		**1**	**3**	**2**	**1**
Narrower spectrum antibiotic		0	3	1	1
Stop antibiotic		1	0	1	0
**Escalation**			**0**		**11**
Escalation/Adaptation			0		4
Escalation usefulness			0		2
Initiation			0		5
**No change after mPCR results**		**44**	**0**	**47**	**16**
**Continuation of antibiotic initiated after suspecting pneumonia**		15	0	20	14
**No new antibiotic**	Continuation of antibiotic initiated before suspecting pneumonia *	27	0	19	2
	**No antibiotic initiation**	2	0	8	0

* antibiotic for a previous infectious episode.

## Data Availability

The datasets used and/or analyzed in the current study can be made available by the corresponding author on reasonable request. The datasets supporting the conclusions are included in the article.

## Supplementary Materials

The following supporting information can be downloaded at: https://www.mdpi.com/article/10.3390/jcm11154366/s1, Table S1. Analytical performance of 125 BioFire^®^ FilmArray^®^ Pneumonia plus Panel compared with culture, irrespective of microbiological thresholds (*n* = 125), Table S2. Analytical performance of BioFire^®^ FilmArray^®^ Pneumonia plus Panel compared with culture, taking into account microbiological thresholds in COVID-19 patients.
