# Supplementary material for: Potential of Multiplex Polymerase Chain Reaction Performed on Protected Telescope Catheter Samples for Early Adaptation of Antimicrobial Therapy in ARDS Patients
Source: J Clin Med. 2022 Jul 27;11(15):4366. doi: 10.3390/jcm11154366 (PMC9369416; doi:10.3390/jcm11154366)
Supplement: Supplementary file 1 [file jcm-11-04366-s001.zip › jcm-1776682-supplementary.pdf]

Table S1 Analytical performance of 125 BioFire® FilmArray® Pneumonia plus Panel compared to culture, irrespective microbiological thresholds (n=125).

Bacterial target	No.of specimens				Performances				Cohen's kappa coefficient
	Culture+/ mPCR+	Culture+/ mPCR-	Culture-/ mPCR+	Culture-/ mPCR-	Se (95% CI), %	Sp (95% CI), %	PPV (95% CI), %	NPV (95% CI), %	
***Acineterobacter calcoaceticus-baumannii* complex**	0	0	0	125	NA	100	NA	100	
***Enterobacter cloacae* complex**	3	0	0	122	100	100	100	100	
Escherichia coli	4	1	0	120	80	100	100	99	
Haemophilus influenzae	0	0	1	124	NA	99	0	100	
Klebsiella aerogenes	3	1	0	121	75	100	100	99	
Klebsiella oxytoca	0	0	0	125	NA	100	NA	100	
***Klebsiella pneumoniae* group**	0	0	0	125	NA	100	NA	100	
Moraxella catarrhalis	1	0	0	124	100	100	100	100	
***Proteus* spp.**	2	1	0	122	67	100	100	99	
Pseudomonas aeruginosa	11	1	1	112	92	99	92	98	
Serratia marcescens	1	0	0	124	100	100	100	100	
Streptococcus pneumoniae	1	0	1	123	100	99	50	100	
Staphylococcus aureus	5	1	3	116	83	97	63	99	
Streptococcus pyogenes	0	0	0	125	NA	100	NA	100	
Streptococcus agalactiae	0	0	2	123	100	99	50	100	
Legionella pneumophila	1	0	1	123	100	99	50	100	
**TOTAL**	**32**	**5**	**9**	**1954**	**86 [75-98]**	**100 [99-100]**	**78 [65-91]**	**100 [100-100]**	**0.82 [0.72-0.91]**

Se, sensibility; Sp, specificity; PPV, positive predictive value; NPV, negative predictive value

Table S2. Analytical performance of BioFire® FilmArray® Pneumonia plus Panel compared to culture, taking into account microbiological thresholds in COVID 19 patients.

Bacterial target	No.of specimens				Performances				Cohen's kappa coefficient
	Culture+/ FA-PP+	Culture+/ FA-PP-	Culture-/ FA-PP+	Culture-/ FA-PP-	Se (95% CI), %	Sp (95% CI), %	PPV (95% CI), %	NPV (95% CI), %	
***Acineterobacter calcoaceticus-baumannii* complex**	0	0	0	106	NA	100	NA	100	
***Enterobacter cloacae* complex**	2	0	1	103	100	99	67	100	
Escherichia coli	2	0	2	102	100	98	50	100	
Haemophilus influenzae	0	0	0	106	NA	100	NA	100	
Klebsiella aerogenes	2	0	1	103	100	99	67	100	
Klebsiella oxytoca	0	0	0	106	NA	100	NA	100	
***Klebsiella pneumoniae* group**	0	0	0	106	NA	100	NA	100	
Moraxella catarrhalis	1	0	0	105	100	100	100	100	
***Proteus* spp.**	2	0	0	104	100	100	100	100	
Pseudomonas aeruginosa	10	0	0	96	100	100	100	100	
Serratia marcescens	1	0	0	105	100	100	100	100	
Streptococcus pneumoniae	0	0	0	106	NA	100	NA	100	
Staphylococcus aureus	2	1	3	100	67	97	40	99	
Streptococcus pyogenes	0	0	0	106	NA	100	NA	100	
Streptococcus agalactiae	0	0	1	105	NA	99	0	100	
Legionella pneumophila	0	0	0	106	NA	100	NA	100	
**TOTAL**	**22**	**1**	**8**	**1669**	**96 [87-100]**	**100 [99-100]**	**73 [58-89]**	**100 [100-100]**	**0.83 [0.72-0.94]**

Se, sensibility; Sp, specificity; PPV, positive predictive value; NPV, negative predictive value
